# Supplementary material for: Effect of Obesity-Linked FTO rs9939609 Variant on Physical Activity and Dietary Patterns in Physically Active Men and Women
Source: J Obes. 2018 Mar 1;2018:7560707. doi: 10.1155/2018/7560707 (PMC5852866; doi:10.1155/2018/7560707)
Supplement: Supplementary Methods — ethnicity information collection. Supplementary Table 1: obesity-related parameters in men and women carrying different risk variants of the FTO rs9939609 single nucleotide polymorphism. Supplementary Table 2: physical activity levels in men and women carrying different risk variants of the FTO rs9939609 single nucleotide polymorphism. Supplementary Table 3: eating behaviour in men and women carrying different risk variants of the FTO rs9939609 single nucleotide polymorphism. [file 7560707.f1.docx]

**Supplementary Information for:**

**Effect of obesity-linked *FTO* rs9939609 variant on physical activity and dietary patterns in physically active men and women**

Nathan R. West^1∞^, James Dorling^2∞^, Alice E. Thackray^2^, Nicola C. Hanson^1^, Samantha E. Decombel^1^, David J. Stensel^2^, Stuart J. Grice^1 3*^

^1^ FitnessGenes, Bicester Innovation Centre, Commerce House, Telford Rd, Bicester OX26 4LD, United Kingdom

^2^ School of Sport, Exercise and Health Sciences, Loughborough University, Loughborough, Leicestershire, LE11 3TU, United Kingdom

^3^ MRC Functional Genomics Unit, Department of Physiology, Anatomy and Genetics, University of Oxford, South Parks Road, Oxford OX1 3PT, United Kingdom

∞ Contributed equally

* Corresponding Author

**Supplementary Methods**

*Ethnicity information collection*

For ethnic background selection, participants were asked which ethnic group they most identified with. Dropdown menus were broken down into (i) Asian: with subgroups including Indian, Pakistani, Bangladeshi, Chinese, any other Asian; (ii) Black: with subgroups including Caribbean, African and Any Other Black background; (iii) White: with subgroups including British, Irish and any other White background; and (iv) Mixed: with subgroups including White and black, White and Asian, White and Chinese, any other Mixed background. An additional text box was provided to allow participants to specify other ethnicities, for example, Hispanic/Latino, Indigenous, Middle Eastern and South American backgrounds. Participants were only included in this study if they identified themselves as non-mixed white descent.

**Supplementary Table 1.** Obesity-related parameters in men and women carrying different risk variants of the *FTO* rs9939609 single nucleotide polymorphism.

| **Characteristic** | ***FTO* rs9939609 genotype** | | | | | **Model 2^a^: Dominant**  **P value (ES/OR)** | **Model 3^b^: Recessive**  **P value (ES/OR)** |
| --- | --- | --- | --- | --- | --- | --- | --- |
|  | **AA** | **AT** | **TT** | **AA/AT** | **AT/TT** |  |  |
| Body mass (kg) | 83.7 (14.7) | 84.4 (16.5) | 82.0 (17.2) | 84.2 (16.0) | 83.3 (16.8) | 0.44 (0.13) | 0.93 (0.02) |
| Body mass index (kg·m^-2^) | 26.8 (3.9) | 26.8 (4.3) | 26.4 (4.3) | 26.8 (4.2) | 26.6 (4.3) | 0.37 (0.09) | 0.79 (0.05) |
| Waist circumference (cm) | 85.2 (9.4) | 84.9 (10.4) | 84.1 (11.0) | 85.0 (10.1) | 84.5 (10.7) | 0.60 (0.09) | 0.88 (0.07) |
| Waist-to-height ratio | 0.48 (0.05) | 0.48 (0.06) | 0.48 (0.06) | 0.48 (0.05) | 0.48 (0.06) | 0.61 (0.03) | 0.68 (0.08) |
| Central obesity^c^ | 6 (7%) | 16 (7%) | 15 (8%) | 22 (7%) | 31 (7%) | 0.70 (0.87) | 0.80 (1.12) |

Values for body mass, body mass index, waist circumference and waist-to-height ratio represent mean (SD) and were analysed using linear mixed models adjusted for age and sex. Values for central obesity represent frequency (%) and were analysed using Chi-square tests. ES, effect size (body mass, body mass index, waist circumference, waist-to-height ratio); OR, odds ratio (central obesity).

^a^ Model 2: dominant model (AA/AT vs. TT)

^b^ Model 3: recessive model (AT/TT vs. AA)

^c^ Central obesity was defined as a waist circumference > 88 cm for women and > 102 cm for men

Note: Statistical analysis for Model 1 (additive genotype model – AA vs. AT vs. TT) is presented in the full manuscript. All statistical analyses using Model 2 (dominant) and Model 3 (recessive) are exploratory.

**Supplementary Table 2.** Physical activity levels in men and women carrying different risk variants of the *FTO* rs9939609 single nucleotide polymorphism.

| **Characteristic** | ***FTO* rs9939609 genotype** | | | | | **Model 2^a^: Dominant**  **P value (ES)** | **Model 3^b^: Recessive**  **P value (ES)** |
| --- | --- | --- | --- | --- | --- | --- | --- |
|  | **AA** | **AT** | **TT** | **AA/AT** | **AT/TT** |  |  |
| Vigorous MET min·week^-1^ | 2808 (1966) | 2751 (1903) | 2407 (1876) | 2768 (1918) | 2589 (1895) | 0.08 (0.19) | 0.29 (0.11) |
| Moderate MET min·week^-1^ | 726 (1069) | 816 (1127) | 654 (874) | 789 (1109) | 740 (1018) | 0.21 (0.13) | >0.99 (0.01) |
| Walking MET min·week^-1^ | 1240 (1242) | 1207 (1345) | 1043 (1192) | 1217 (1313) | 1130 (1276) | 0.17 (0.14) | 0.32 (0.09) |
| Total MET min·week^-1^ | 4774 (3125) | 4774 (3193) | 4104 (2795) | 4774 (3167) | 4459 (3027) | 0.03 (0.22)** | 0.28 (0.10) |

Values are mean (SD) for *n* = 408. Comparisons were made using linear mixed models adjusted for age and sex. ES, effect size.

^a^ Model 2: dominant model (AA/AT vs. TT)

^b^ Model 3: recessive model (AT/TT vs. AA)

** Significant difference between AA/AT and TT *FTO* rs9939609 genotype (linear mixed model P < 0.05)

Note: Statistical analysis for Model 1 (additive genotype model – AA vs. AT vs. TT) is presented in the full manuscript. All statistical analyses using Model 2 (dominant) and Model 3 (recessive) are exploratory.

**Supplementary Table 3.** Eating behaviour in men and women carrying different risk variants of the *FTO* rs9939609 single nucleotide polymorphism.

| **Characteristic** | ***FTO* rs9939609 genotype** | | | | | **Model 2^a^: Dominant**  **P value (ES)** | **Model 3^b^:**  **Recessive**  **P value (ES)** |
| --- | --- | --- | --- | --- | --- | --- | --- |
|  | **AA** | **AT** | **TT** | **AA/AT** | **AT/TT** |  |  |
| Cognitive restraint score | 13 (4) | 12 (4) | 11 (4) | 12 (4) | 11 (4) | 0.12 (0.14) | 0.01 (0.28)*** |
| Disinhibition score | 6 (3) | 7 (4) | 6 (4) | 6 (4) | 6 (4) | 0.32 (0.07) | 0.60 (0.08) |
| Hunger score | 5 (3) | 5 (4) | 5 (4) | 5 (4) | 5 (4) | 0.99 (0.02) | 0.69 (0.01) |

Values are mean (SD) for *n* = 528. Comparisons were made using linear mixed models adjusted for age and sex. ES, effect size.

^a^ Model 2: dominant model (AA/AT vs. TT)

^b^ Model 3: recessive model (AT/TT vs. AA)

*** Significant difference between AT/TT and AA *FTO* rs9939609 genotype (linear mixed model P < 0.05)

Note: Statistical analysis for Model 1 (additive genotype model – AA vs. AT vs. TT) is presented in the full manuscript. All statistical analyses using Model 2 (dominant) and Model 3 (recessive) are exploratory.
